# Potential Applications of RNase P Ribozyme Against Hepatitis B Virus

**DOI:** 10.3390/molecules30183725

**Published:** 2025-09-12

**Authors:** Thomas Sorrell, Yujun Liu, Fenyong Liu

**Affiliations:** 1 School of Public Health, University of California, Berkeley, CA 94720, USA; 2 Department of Molecular and Cell Biology, University of California, Berkeley, CA 94720, USA; 3 Program in Comparative Biochemistry, University of California, Berkeley, CA 94720, USA

**Keywords:** antisense, antiviral therapy, gene targeting, gene therapy, hepatitis B virus, RNase P, ribozyme

## Abstract

Nucleic acid-based gene-interfering molecules, such as antisense oligonucleotides, ribozymes, and small interfering RNA (siRNA), represent exciting gene-targeting agents for therapeutic applications. RNase P ribozymes derived from M1 RNA, the catalytic RNA subunit of RNase P in *Escherichia coli*, have shown great promise as a novel nucleic acid-based gene interference approach to modulate gene expression. When M1 RNA is covalently linked to a guide sequence (GS), it can be engineered into a sequence-specific endonuclease M1GS ribozyme, which can hydrolyze any mRNA that base-pairs with the guide sequence. M1GS activity enhancement has been achieved through an in vitro selection process that introduced mutations into M1 RNA. This selection process generated ribozyme variants with improved cleavage efficiency and substrate affinity. Hepatitis B virus (HBV) chronically infects more than 250 million people worldwide and is the leading cause of cirrhosis and liver cancer globally. Current FDA-approved drugs cannot completely eliminate HBV chronic infections. RNase P ribozymes have recently been demonstrated to effectively inhibit HBV gene expression and replication in human cells. This review summarizes the recent progress in using RNase P ribozymes to inhibit HBV infection and discusses prospects for developing engineered RNase P ribozymes for therapeutic applications against HBV infection and associated diseases.

## 1. Introduction

Hepatitis B virus (HBV) chronically infects more than 250 million people worldwide and remains the leading cause of viral liver disease [1,2,3,4]. Although antiviral therapies have been effective in suppressing viral replication and symptoms, current treatments are limited by their inability to eliminate chronic infections [5]. Targeted gene regulation using nucleic acid-based strategies has emerged not only as a promising candidate for therapeutic intervention but also as a driving force in the advancement of molecular biology and drug development. Among these strategies are antisense oligonucleotides, ribozymes, RNA interference (RNAi), and the genome editing technology based on Clustered Regularly Interspaced Short Palindromic Repeats (CRISPR)/CRISPR-associated protein (Cas) RNA-guided nuclease systems [6,7,8]. Each strategy operates distinctly, from steric hindrance in translation to site-specific cleavage of the target RNA or DNA molecules, providing multiple levels for targeting gene expression.

The M1GS ribozyme, which fuses the catalytic RNA subunit (M1 RNA) of ribonuclease P (RNase P) from *Escherichia coli*, with a guide sequence (GS), offers a distinct and unique RNA-targeting nucleic acid-based interference strategy [9]. By reshaping a target mRNA into a pre-tRNA-like substrate, the endogenous structure-based cleavage mechanism of RNase P M1 RNA can be redirected into a sequence-specific manner [10,11]. M1GSs are highly engineerable through the design of GS with higher binding affinity and the selection of M1GS variants with enhanced catalytic efficiency. A single M1GS can cleave multiple copies of target mRNA, and the risk of genomic alteration is avoided due to its activity exclusive to the mRNA level [9]. In this review, we will discuss the potential of M1GS for treating HBV infection.

## 2. Hepatitis B Virus

**Figure 1:**
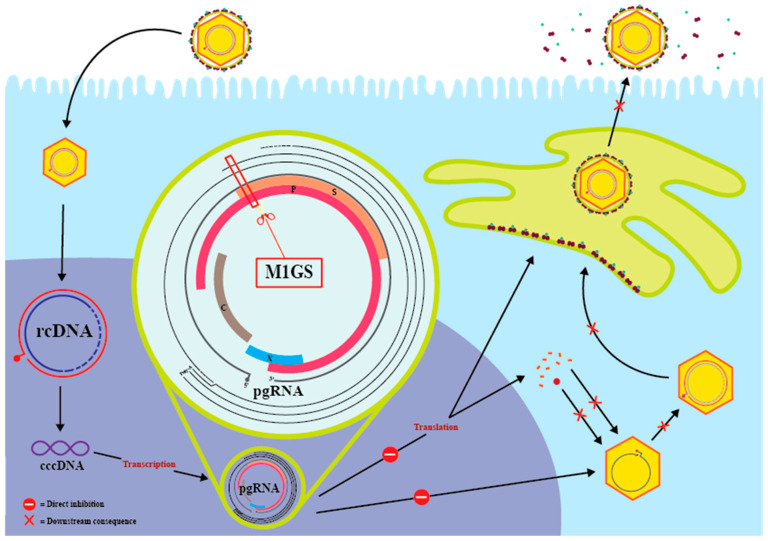
Schematic of the HBV lifecycle and inhibition by M1GS. HBV relaxed circular DNA (rcDNA) is converted into covalently closed circular DNA (cccDNA) in the nucleus, which serves as a template for transcription of pregenomic RNA (pgRNA). pgRNA has dual roles as the template for reverse transcription into rcDNA and as mRNA for viral proteins (polymerase, core, surface, and HBx proteins). M1GS targets overlapping regions of HBV mRNA and pgRNA, resulting in their cleavage and degradation. M1GS directly inhibits translation and reverse transcription, which in turn prevents cccDNA replenishment and the production of new virions.

HBV is a hepatotropic DNA virus that infects more than 250 million people chronically worldwide and is the leading viral cause of liver disease, including hepatocellular carcinoma [1,3,4]. HBV acquires its lipid bilayer from the host hepatocyte, in which small, middle, and large-sized surface antigens are embedded [2]. Beneath the envelope lies its nucleocapsid, formed by 120 core proteins that enclose the 3.2 kb relaxed circular DNA (rcDNA) genome [12]. Viruses, constrained by the capsid’s volume, are often characterized by their abundance of overlapping open reading frames (ORFs) [13]. In HBV, there are four ORFs encoding polymerase (P), surface (S), precore/core (C), and X, and approximately 52% of HBV’s nucleotides encode more than one viral protein, a genomic arrangement illustrated in Figure 1 [12,14]. Overlapping ORFs provide the virus with genomic efficiency and can significantly enhance its evolution rate, with a single point mutation capable of affecting multiple proteins or creating new ORFs entirely [13,15]. This arrangement, although advantageous for the virus, can be converted into a vulnerability by nucleic acid-based gene-interfering molecules, such as M1GS, which target overlaps to suppress the expression of multiple essential proteins simultaneously.

After the rcDNA enters the hepatocyte’s nucleus, host DNA repair machinery converts it into covalently closed circular DNA (cccDNA) [2,12] (Figure 1). The viral cccDNA, a stable nuclear minichromosome, serves as a reservoir for chronic HBV infection by utilizing endogenous epigenetic pathways to avoid degradation and evade detection by the host immune system [16,17]. During cellular stress or immunosuppression, this epigenetic repression of cccDNA is reversed, allowing host RNA Pol II to transcribe cccDNA into pre-genomic RNA (pgRNA) [16,17]. The pgRNA serves dual roles: as a template for reverse transcription into rcDNA and as mRNA for HBV polymerase and core proteins [2,12,18] (Figure 1). Meanwhile, rcDNA-containing nucleocapsids may either be packaged into virions or recycled back to the nucleus. HBV DNA can be recycled as rcDNA, where it can form cccDNA, or as double-stranded linear DNA (dslDNA), which can integrate into the host DNA [12,19]. When integrating into the host genome, HBV DNA can activate oncogenes or silence tumor suppressor genes, leading to the development of hepatocellular carcinoma [2].

Current treatments target HBV at three levels: (i) the protein level, (ii) the enzymatic level, and (iii) the DNA level [20]. HBV vaccines contain surface antigens, HBsAg, which train the adaptive immune system to recognize and respond to new infections [2]. Nucleos(t)ide analogs (NAs), such as tenofovir or entecavir, inhibit HBV polymerase by competing with nucleotides [12,20]. Incorporated NAs lead to chain termination during the reverse transcription of pgRNA into rcDNA within the nucleocapsid, blocking the production of new virions [21]. IFN-α and other interferons induce cytidine deaminases that create abasic sites, triggering DNA repair mechanisms and leading to partial degradation of cccDNA [22,23]. These treatments are limited by toxicity, their preventive or suppressive nature, and their inability to eliminate cccDNA completely. As long as cccDNA is present, even patients who have received treatment remain at risk of developing liver cancer [24]. This shifts the clinical focus to targeting pgRNA, a link to both cccDNA and all HBV components. Targeting pgRNA can disrupt translation of polymerase, core, surface, and HBx proteins, reverse transcription into rcDNA, replenishment of cccDNA, and production of new virions [2]. Reducing pgRNA with nucleic acid-based gene targeting agents represents an effective strategy for targeting HBV at the RNA level.

## 3. Nucleic Acid-Based Gene Targeting Agents for Therapy of HBV Infections and Diseases

Among the nucleic acid-based gene targeting strategies, RNA interference (RNAi) and CRISPR-Cas9 systems have remained popular options. RNAi, similar to M1GS, offers multi-turnover potential [6,7]. Through the recruitment of cellular machinery, small interfering RNA (siRNA) directs the degradation of mRNA substrates, enabling gene interference at the mRNA level [25] (Table 1). Delivery to hepatocytes represents a significant challenge for the treatment of HBV infection using RNAi. Many strategies address this through hepatocyte-targeted membrane-lytic peptides (NAG-MLP) [26] (Table 1). The therapeutic RNA is attached to a liver-specific ligand that induces endocytosis. The membrane-lytic peptide then enables RNA to escape from the endosome into the cytoplasm, where it can interact with cellular machinery (RISC) to cleave HBV transcripts [27]. This approach has been largely successful in preclinical models. The key limitation of RNAi therapy is that siRNA undergoes rapid clearance, necessitating repeated dosing to maintain suppression and thereby failing to address the chronic nature of HBV infection [27]. Determining the safety profile, long-term effects, and dosage will require data from clinical studies, delaying the transition to clinical application for chronic HBV treatment. Long-term delivery of RNAi using lentiviral, adenoviral, and adeno-associated viral vectors (AAVs) has been explored for sustained expression of therapeutic siRNAs [26]. However, studies demonstrate that at high siRNA concentrations, RNAi machinery can become overwhelmed, leading to off-target effects [28,29]. As a result, long-term efficacy, avoidance of saturation, and non-toxic tissue-specific delivery have been significant focuses of ongoing research.

The application of CRISPR-Cas9 systems in treating HBV has been primarily focused on the cleavage of cccDNA (Table 1). This approach has been largely successful in depleting cccDNA reservoirs; however, concerns about off-target cleavage remain [30]. Current studies have reduced off-target cleavage by exploring alternative Cas9 variants, such as *Streptococcus thermophilus* Cas9 (StCas9), which requires a longer PAM sequence, thereby offering higher specificity [30]. The higher precision comes at the cost of reduced targeting flexibility, increasing the likelihood that HBV variants evade detection and cleavage. In contrast to RNAi and CRISPR-Cas9 systems, meganucleases, zinc finger nucleases (ZFNs), and transcription activator-like effector nucleases (TALENs) rely on direct protein–DNA interaction for site-specific cleavage (Table 1) [26]. Meganucleases combine DNA binding and cleavage into a single molecule, while ZFNs and TALENs combine separate DNA-binding domains with a FokI nuclease [31]. These systems have been utilized in antiviral strategies due to their high specificity and relatively low off-target effects. However, their complex design, limited flexibility for multiple target sites, delivery challenges, and sensitivity to DNA methylation represent major setbacks in their clinical applications [31].

M1GS represents a highly programmable alternative to current nucleic acid-based gene interference strategies [9]. Other ribozyme platforms, such as hammerhead and hairpin ribozymes, have been explored for HBV inhibition [6,7]. For example, hairpin ribozymes targeting the core (C) gene were successful in cleaving HBV transcripts in vitro [32]. Both hammerhead and hairpin ribozymes require a conserved sequence at the cleavage site, limiting their design flexibility. In contrast, M1GS’s structure-based recognition allows sequence-specific cleavage as long as correct base pairing and folding occur [10,33]. Significantly, because HBV’s genome contains overlapping reading frames, a single pgRNA-targeting ribozyme construct, whether hammerhead, hairpin, or M1GS, can simultaneously disrupt translation, transcription, and recycling of rcDNA into cccDNA. In this review, we discuss the potential application of this multi-faceted approach to interfere with HBV’s life cycle, showcasing the viability of M1GS as a potent antiviral against HBV infection.

**Table 1:** Clinical and preclinical advances in nucleic acid-based and gene-editing therapeutic strategies against HBV.

Approach	Target	Specificity	Delivery Challenges	Clinical Stage	Results	Key Advantages	Key Limitations	Reference
Meganucleases	P ORF	High	Used lipid nanoparticles for mRNA delivery. Need for efficient liver targeting.	Preclinical	Resulted in ~85% reduction in cccDNA and reduced HBV DNA by 80%	Highly specific DNA recognition and cleavage. Compact size (mRNA delivery) allows for various delivery methods. Can be engineered to enhance specificity, thereby reducing off-target effects.	Complex engineering process and potential for DNA integration at cut sites. Efficacy depends on optimization and limited flexibility in target site selection.	[34]
ZFNs	P, C, and X ORFs	Generally high, but can vary	Two-component system limits packaging capacity (requires two scAAV vectors). There is a need for efficient liver targeting and co-delivery of both components.	Preclinical	Complete inhibition of HBV DNA replication and production of virions for at least 2 weeks after treatment.	Highly specific DNA recognition and cleavage. Customizable specificity and flexible target selection through modifying/swapping zinc finger modules.	Complex design process, potential off-target effects, cytotoxicity (ZFN2), varying efficacy across HBV genotypes (may require optimization for variants). Properties of zinc finger domains result in a preference for targeting sequences containing GNN, ANN, or CNN triplets.	[35,36]
TALENs	Conserved regions of P and C ORFs of multiple HBV genotypes (A-D)	High	Large size limits delivered options (hydrodynamic injection used in this study). Adenovirus/AAV vectors can also be used for hepatic delivery.	Preclinical	Reduced core protein expression by ~79% and HBV DNA levels by ~73%. Decreased cccDNA by ~10–20%.	Highly specific DNA recognition and cleavage. Shown to be synergistic when combined with IFN-α, an HBV antiviral, and was effective against multiple HBV genotypes.	Large size complicates efficient delivery to hepatocytes. It requires two TALENs to bind near each other. Efficacy varies depending on target sequences (potential for nucleosome positioning to interfere with cccDNA binding)	[37]
ASOs (Bepirovirsen)	A 20-nucleotide sequence present in all HBV mRNA and pgRNA	High	2′-O-methoxyethyl modification to enhance stability against nuclease degradation. Subcutaneous injection of lipid nanoparticles.	Phase III (NCT04449029)	9–10% achieved primary endpoint (HBsAg loss and undetectable HBV DNA at week 24 post-treatment)	Proven to be effective in combination with other antiviral NA therapies. Rapid viral response was observed in some patients.	Limited efficacy, with only 9–10% of patients achieving the primary endpoint. Weekly injections are required, with a likely need for repeated dosages to achieve a functional cure.	[38,39]
RNAi (JNJ-3989)	Targets X and S ORFs	High	GalNAc-conjugated siRNA for targeted liver delivery is administered through subcutaneous injection. The main delivery challenge is maintaining therapeutic levels over time.	Phase II (NCT04129554)	JNJ-3989 + Bersacapavir (HBV core protein inhibitor) + NA reduced HBsAg by ~98% at the end of treatment compared to ~13% in the control (NA only). At week 24 post-treatment, 81.5% of patients maintained at least a 90% reduction.	The use of GalNAc made these siRNAs highly tissue-specific. RNAi generally offers high specificity and has been proven effective in combination with other antivirals. The small size of RNAi allows for various delivery methods	A functional cure was not achieved in this study. Suppression was not completely maintained in all patients post-treatment. High concentrations of RNAi can potentially cause off-target effects, which may partly account for the grade 3 or 4 adverse events observed in this study (~15% of patients)	[28,29,40,41]
CRISPR/Cas9 (Non-cleavage Base Editing)	P and S ORFs	High	Lentiviral transduction in cell culture. Large size makes delivery challenging in vivo.	Preclinical	HBV DNA decreased by more than 60%. ~25%–35% C→T conversion at target sites for cccDNA and ~35%–80% C→T conversion at target sites for integrated HBV DNA.	Avoids DSBs and potential chromosomal translocations that could be caused by WT CRISPR/Cas9 systems cleaving integrated HBV DNA. Capable of targeting both cccDNA and integrated HBV DNA, allowing for permanent inactivation of HBV.	The large size of Cas9 Base editors makes in vivo delivery challenging. They share the same off-target challenges as WT Cas9, with the risk of generating nonsense mutations in the host genome. The potential effects of truncated HBV proteins are unknown.	[42]
M1GS	Overlapping region of pgRNA, S mRNA, and pre-S/L mRNA.	High	Plasmid transfection in cell culture. Large size makes delivery challenging in vivo.	Preclinical	~82% reduction of HBV RNA, ~80% reduction in HBsAG and HBeAg, and ~300-fold decrease in HBV DNA.	High specificity is achieved due to structure recognition during cleavage. Mismatches that might be tolerated in other sequence-based systems are avoided. M1GS are highly modular and can be optimized through in vitro selection. This study utilized WT M1 RNA, allowing for efficacy improvement through an in vitro selection process.	The large size of M1GS makes delivery in vivo challenging. Tissue specificity, a challenge in M1GS delivery, can be addressed through tailored strategies; however, this compromises the highly programmable nature and flexibility that make M1GS a desirable therapeutic.	[43]

## 4. RNase P and Its Catalytic RNA

Ribonuclease P (RNase P) is a holoenzyme found in all domains of life, responsible for cleaving the 5′ leader sequence from tRNA precursors and other small RNAs [10,44,45]. In *Escherichia coli*, RNase P is composed of M1 RNA, where catalytic activity takes place, and a stabilizing protein component. RNase P has one protein component in bacteria, typically 4 in Archaea, and up to 10 in Eukaryotes [10,46].

M1GS utilizes M1 RNA from *Escherichia coli* RNase P, which is 377 nucleotides in length [11]. RNase P requires both the RNA and protein subunits in vivo. The protein subunit is responsible for stabilizing the structure and folding of M1 RNA during its active formation, thereby favoring pre-tRNA over mature tRNA by improving binding affinity and possibly pre-organizing metal ion binding sites [47,48,49,50,51,52,53,54,55]. These roles have been supported through the structural analysis of M1 RNA–protein interaction through crystallography and cryo-EM, as well as mutational and phylogenetic studies [10,11,44,56,57]. Human RNase P consists of H1 RNA and at least 10 protein subunits, functionally analogous to the *Escherichia coli* C5 protein subunit; however, the precise function of each subunit remains a key objective for future studies [46,58].

In a groundbreaking study of M1 RNA, it was demonstrated that M1 RNA is capable of cleaving pre-tRNA in the absence of the protein subunit under high $Mg^{2+}$ concentrations (i.e., ~100 mM) in vitro [49]. The positively charged ions shielded the negatively charged phosphate backbone of the RNA, thereby reducing electrostatic repulsion and allowing M1 RNA to fold into its active structure [47,48,49,50,51,52,53,54,55]. This finding was pivotal not only in confirming that M1 RNA itself possesses catalytic activity but also in laying the groundwork for engineering RNA-only constructs, thereby shifting the focus to RNA design.

## 5. RNase P Substrate Recognition and Engineering of Gene-Targeting Ribozymes from RNase P RNA

### 5.1. Structural Basis of Substrate Recognition

**Figure 2:**
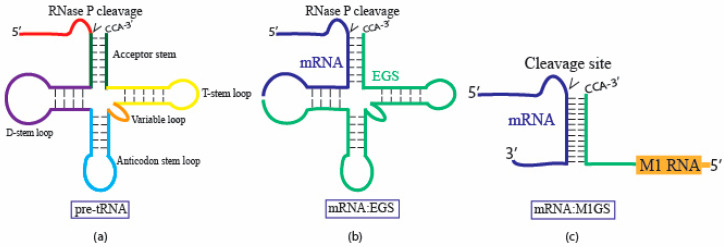
Substrates for RNase P and M1GS RNA. (**a**) The natural pre-tRNA substrate contains structural elements including the acceptor stem, T-stem loop, D-stem loop, variable loop, and anticodon stem loop. RNase P cleaves at the junction between the acceptor stem and the CCA 3′ end. (**b**) External guide sequence (EGS) base pairs with the target mRNA to fold into a pre-tRNA-like structure, enabling human RNase P-mediated cleavage. (**c**) M1GS associates with a target mRNA through a covalently linked guide sequence, enabling M1 RNA-mediated cleavage.

RNase P acts on substrates that share structural motifs such as the T-stem/loop, elements resembling the acceptor stem, and unpaired 5′ leaders, which are highlighted in Figure 2. By detecting structure rather than a specific sequence, RNase P can cleave a variety of RNAs, including pre-tRNA and 4.5S RNA precursors [10,11,44,45]. Early studies expanded upon this concept by minimizing the substrate to include only the acceptor stem, T stem/loop, and the 3′ CCA sequence [59,60]. RNase P recognized this structure and cleaved the 5′ leader sequence, demonstrating that the complete pre-tRNA structure is not necessary as long as these structures remain (Figure 2a). This insight led to the development of external guide sequences (EGS), short RNAs that detect and bind to target RNAs through Watson–Crick interactions [59,61]. These interactions reshape the RNA substrate into a recognizable pre-tRNA structure that RNase P can recognize and cleave (Figure 2b). EGS utilizes RNase P for both substrate recognition and cleavage. As a result, specificity can be easily reprogrammed by altering only the EGS, rather than the pre-existing cellular mechanism, making EGS a highly convenient and adaptable tool for nucleic acid-based gene interference [9,10,11].

### 5.2. Engineering Principles for M1GS Ribozymes

EGS has been applied to HBV, where EGS constructs were designed to target the overlapping region of the S mRNA, pre-S/l mRNA, and pregenomic RNA (pgRNA) using *Salmonella*-mediated delivery [62,63]. The first study employed an engineered EGS variant in vitro, resulting in a ~97% reduction in HBV RNA and proteins, and a ~6000-fold decrease in capsid-associated HBV DNA in cultured hepatocytes [63]. A follow-up study expanded on these findings by using a different variant in a mouse model. Delivery of the EGS via *Salmonella* into the mouse liver resulted in a ~95% reduction in HBV gene expression and a ~200,000-fold decrease in viral DNA levels in the liver and sera of the treated mice [62]. These studies confirmed the antiviral activity of EGS in both in vitro and in vivo settings. RNase P is expressed and active at all stages of the cell cycle due to its role in tRNA maturation, making EGS effective regardless of the stage of the cell cycle [10,44,45]. However, EGSs are limited by their reliance on endogenous RNase P recruitment.

**Figure 3:**
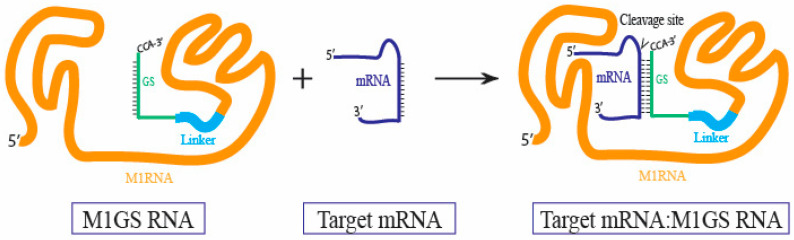
M1GS binds to a target mRNA. The M1GS RNA contains an *Escherichia coli* M1 RNA catalytic domain linked to a guide sequence (GS) via a short linker. The GS base pairs with a complementary region in the target mRNA, inducing the target to fold into a pre-tRNA-like structure containing an acceptor stem, T-stem loop, and other structural features shown in Figure 2a. This mimicry enables the catalytic M1 RNA domain to recognize the substrate, resulting in site-specific cleavage.

Shared RNA structure of EGS and M1 RNA led to the development of M1GS, which combines the EGS substrate recognition with RNase P’s catalytic M1 RNA (Figure 2c and Figure 3). As illustrated in Figure 3, through the linkage of a guide sequence (GS) to M1 RNA, the ribozyme forces proximity of the catalytic M1 RNA to the target site, addressing the issue of EGS’s reliance on host RNase P. Research has revealed that M1GS exhibits higher cleavage efficiency and substrate binding at low $Mg^{2+}$ concentrations, more accurately reflecting in vivo conditions, compared to unlinked M1 RNA and EGS [33,55,64,65]. M1GS constructs delivered into human cells must rely on human RNase P proteins instead of the C5 bacterial protein. It is believed that the protein subunits of human RNase P bind to regions of M1 RNA recognized by C5, providing a compensatory interaction [46]. Studies have demonstrated that C5 proteins enhance M1GS activity 30-fold compared to purified human RNase P proteins, which improve activity 5-fold [65,66,67]. The fully RNA-composed structure of M1GS presents opportunities for optimizing compatibility with human RNase P proteins.

## 6. In Vitro Evolution of RNase P Ribozyme with Improved Gene Targeting Activity

**Figure 4:**
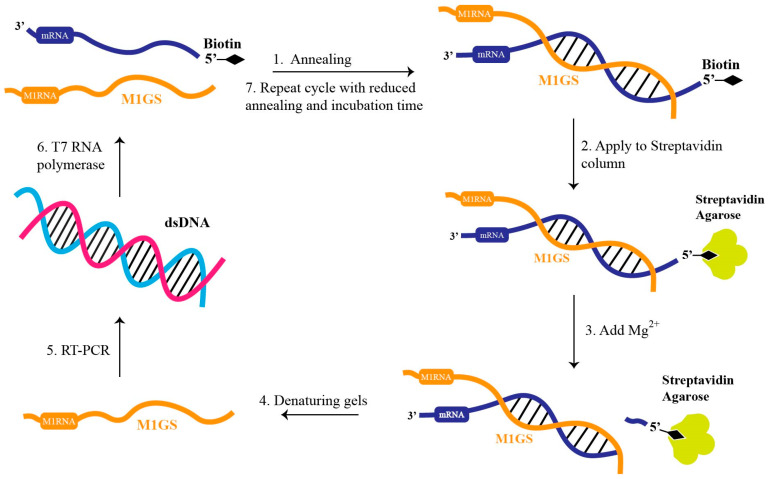
In vitro selection procedure to engineer RNase P ribozyme (M1GS) variants that cleave mRNA targets more efficiently. Under these in vitro conditions, high $Mg^{2+}$ concentrations allow M1 RNA to function without its protein cofactor. A randomized ribozyme pool is annealed to a biotinylated target mRNA at 37 °C (step 1), bound to a streptavidin column (step 2), and activated by $Mg^{2+}$ to cleave the target (step 3). Active ribozymes are recovered from denaturing gels (step 4), reverse-transcribed, and PCR-amplified (step 5), then transcribed back into M1GS RNA (step 6), and reintroduced into subsequent rounds with reduced annealing and incubation times to increase selection stringency (step 7).

M1GS’s catalytic efficiency and binding affinity can be optimized through an in vitro evolution/selection process [68,69,70] (Figure 4). This process, as illustrated in Figure 4, begins by generating a randomized M1 ribozyme pool with mutations within the M1 RNA and conserved regions [71,72]. These variants are then given time to anneal with a 5′-biotinylated target mRNA substrate (Figure 4). The annealed M1GS-mRNA complexes are passed through a streptavidin column that tightly binds to the biotinylated mRNA. Misfolded M1GS or those that otherwise fail to bind to the mRNA substrate pass through this column [71,72]. $Mg^{2+}$ ions are then introduced, allowing the catalytically active M1GS to cleave the mRNA substrate, releasing it from the column (Figure 4). Captured ribozymes are then isolated through gel electrophoresis, reverse-transcribed, and subjected to PCR amplification, followed by further rounds of increasingly stringent conditions with reduced annealing and incubation times, until no additional improvements in cleavage efficiency are observed (Figure 4) [71,72].

In vitro selection processes [71,72] have been utilized to generate M1GS for the treatment of herpes simplex virus 1 (HSV-1) infection with increased catalytic and binding affinity, making it more suitable for clinical applications [71,73]. Compared to wild-type M1GS, these variants exhibited a 20-fold increase in catalytic efficiency and a more than 50-fold increase in binding affinity for an HSV-1 thymidine kinase (TK) mRNA substrate [71]. These variants were then tested in HSV-1-infected cells, resulting in a 99% reduction in TK mRNA levels and a 98% reduction in TK protein levels [71]. This study confirmed that the improved M1GS generated by the in vitro selection process functions in a cellular context, paving the way for future research on optimizing M1GS tailored for any mRNA target.

## 7. Inhibition of HBV Gene Expression and Growth by M1GS RNA

Chronic HBV infection is sustained through covalently closed circular DNA (cccDNA) in the nucleus of infected hepatocytes [16,17]. Current treatments suppress viral replication but do not effectively eliminate the cccDNA reservoir [24]. As a result, cccDNA continues to serve as a template for pregenomic RNA (pgRNA), maintaining the risk of hepatocellular carcinoma and reactivation. The pgRNA is not only the transcript for viral proteins but also acts as a template for reverse transcription into rcDNA (Figure 1) [12,18]. The rcDNA can be recycled back into the nucleus to replenish the cccDNA pool or integrate itself into the host genome [2]. Integration into the host genome can activate oncogenes or inactivate tumor suppressor genes, leading to the development of hepatocellular carcinoma [12,19]. M1GS offers a novel approach by targeting overlapping regions of pgRNA and other HBV transcripts (Figure 1). Cleavage at these sites not only blocks translation but also inhibits reverse transcription and recycling of rcDNA back into cccDNA, the driver of chronic infection.

RNase P ribozymes targeting HBV were investigated by using M1GS targeting the overlapping region of HBV S mRNA, pre-S/L mRNA, and pregenomic RNA (pgRNA) [43]. Researchers began by using dimethyl sulfate (DMS) mapping in HepG2.2.15 cells to identify single-stranded, ribozyme-accessible sites in the S mRNA and pgRNA regions [43]. After determining the accessible area, researchers engineered three M1GS constructs: M1-S-A (an active ribozyme), M1-S-I (an inactive ribozyme with a mutation in the catalytic site), and M1-P (a non-HBV-targeting ribozyme). HepG2.2.15 cells were transfected with these ribozymes, and Northern blot analysis was utilized to determine HBV RNA levels. In cells expressing M1-S-A, there was an ~82% reduction in HBV RNA (M1-S-I < 10%, M1-S-P ~0%) [43]. ELISA assays indicated an ~80% reduction in HBsAg and HBeAg, and qPCR analysis recorded a ~300-fold decrease in capsid-associated HBV DNA [43]. These findings demonstrate the efficacy of M1GS in reducing HBV transcripts, proteins, and DNA in cultured human cells. These findings support the mechanism of action by blocking cccDNA replenishment by targeting pgRNA, which interferes with the steps required to recycle rcDNA into the cccDNA reservoir, a key process in sustaining chronic HBV infection. It is important to note that this level of suppression was achieved using wild-type M1 RNA. Future improvements can be obtained by applying an in vitro selection process to generate M1GS variants with enhanced catalytic efficiency and substrate affinity.

## 8. Advantages and Disadvantages of M1GS RNAs

M1GS stands out from other therapeutic approaches due to several unique strengths. For example, classical antisense oligonucleotides rely on host RNase H to degrade RNA-DNA hybrids while carrying the risk of non-target cleavage due to RNase H’s tolerance to imperfect base pairing [6,7,74]. In comparison, M1GS utilizes Watson–Crick base pairing to remodel its target into a pre-tRNA-like structure. Due to substrate recognition being based on structure, mismatches that might be tolerated in sequence-based systems are avoided, as only correctly base-paired GS-RNA duplexes can fold into the pre-tRNA structure required for recognition and cleavage by M1GS [9,75]. Hammerhead and hairpin ribozymes are also RNA-based and share similarities with the M1GS; however, they require a specific nucleotide sequence (GUX) in the target mRNA, which limits their applicability [33,76,77]. M1GS, derived from *Escherichia coli* M1 RNA component of RNase P, acts catalytically and irreversibly. Although M1 RNA typically functions with the bacterial C5 protein cofactor, it can also interact with human RNase P protein subunits, and such compensatory interactions have been shown to enhance M1GS activity in human cells [46,65,66,67]. Together, these features make M1GS a compelling candidate for gene-targeting antiviral therapy.

Significant challenges remain when considering the therapeutic application of M1GS. One concern is the limited research available to fully understand the potential unintended effects of M1GS overexpression on cellular physiology. If expressed at high levels, M1GS could interfere with human RNase P and its associated pathways [46]. Additionally, M1GS and other RNA-based strategies are susceptible to degradation. While different strategies implement chemical modification, such as the addition of a 2′ hydroxyl or phosphorothioate linkage to resist endonuclease activity, such modification can disrupt the precise folding necessary for M1GS due to its reliance on secondary and tertiary structures for catalytic function [7,78]. Finally, the relatively large size of M1GS RNA (~400 nucleotides) poses a significant challenge for delivery and synthesis. In contrast to RNAi-based approaches, which benefit from established delivery platforms such as lipid nanoparticles and polymeric matrices, the size of M1GS may make these methods less effective [33,79]. As a result, the successful delivery of M1GS requires the use of viral vectors or the development of new delivery strategies tailored to M1GS.

## 9. Future Direction and Challenges

First, future directions could involve enhancing the compatibility of M1GS with human RNase P proteins and optimizing the catalytic core of M1 RNA itself. This can be achieved through an in vitro selection process (Figure 3) by selecting for variants with increased catalytic and binding affinities from a pool of M1GS variants generated by mutagenizing the M1 RNA and its conserved regions [71,72]. This in vitro selection process can be adapted to optimize compatibility with human RNase P proteins by conducting selection in the presence of purified human RNase P protein cofactors.

Second, future studies could investigate the delivery of M1GS. Previous studies have successfully utilized attenuated *Salmonella* strains to deliver EGS constructs to the liver, achieving tissue-specific gene silencing [62,63]. This bacterial vector approach has been confirmed to be effective for M1GS delivery in previous studies targeting viral infection [33,80,81,82]. Although attenuated Salmonella strains can activate immune pathways that may interfere with therapeutic efficacy, this limitation has been addressed through the development of strains such as SL101, which exhibits high gene transfer activity and low cytotoxicity and pathogenicity in vivo [80]. SL101 has been successfully used for M1GS delivery; however, its efficacy has not yet been evaluated in combination with other antiviral agents. Retroviral-based strategies have been successful in delivering M1GS both in vivo and in vitro [26,83]. Future studies could expand upon these strategies by investigating the application of liver tropic virus-based vectors and non-virus-based vectors to enhance hepatocyte specificity for targeted M1GS expression.

Recent advances in non-viral delivery platforms such as engineered lipid nanoparticles (LNPs) and exosome-based carriers have demonstrated the capacity to deliver larger RNA molecules, often several kilobases in length, far exceeding the approximately 400-nucleotide length of M1GS [84,85]. In a recent study, researchers discovered that optimizing the alkyl tail length of ionizable lipids in LNPs significantly enhanced the delivery efficiency of mRNA cargo of varying sizes [84]. Notably, LNPs formulated with C9-200, featuring a shortened 9-carbon alkyl tail, considerably improved the delivery of large Cas9 mRNA (~4.5 kb) [84]. This improvement was demonstrated through a threefold increase in gene editing efficiency, as measured by the number of indels compared to the standard C12-200 LNP [84]. In another study, researchers developed hybrid exosomes by fusing two types of nanoparticles: exosomes and cubosomes [85]. Exosomes are naturally occurring, but they struggle to carry large therapeutic molecules, such as mRNA. Cubosomes, in contrast, are synthetic nanoparticles engineered to encapsulate larger molecules, but they struggle to cross biological barriers. Researchers fused these two nanoparticles to create hybrid exosomes, which, when tested in a blood–brain barrier model, demonstrated a two-fold increase in permeability for both large proteins and mRNA compared to cubosomes alone [85]. Adapting these platforms for M1GS could provide the same advantages observed in these studies, including improved encapsulation efficiency, protection from degradation, and enhanced delivery across biological barriers.

Third, future studies will focus on improving the safety profile of M1GS in vivo. For example, the pharmacokinetic properties of M1GS require investigation. The distribution and turnover of M1GS in vivo should be studied. Substantial progress has recently been made in reducing the immunogenicity and immunotoxicity of RNA molecules by incorporating various modifications into chemically or T7 RNA polymerase-synthesized RNA molecules (e.g., 1-methyl-pseudouridine) and developing nanoparticles for exogenous RNA delivery [86,87]. Future studies will be conducted to determine if M1GS, when synthesized with these modifications and assembled with improved nanoparticles, exhibits enhanced pharmacokinetic properties in vivo.

To improve M1GS specificity and reduce its potential off-target effects, ribozymes with enhanced sequence specificity and catalytic activity can be constructed by engineering the M1GS substrate and catalytic domains, followed by refinement through in vitro selection procedures (Figure 4). To address potential viral escape from M1GS targeting, multiple ribozymes should be constructed to target various conserved regions of HBV pgRNA. Multiplex targeting by several M1GS RNAs simultaneously at different conserved HBV pgRNA sequences is expected to reduce the emergence of viral mutants that escape M1GS targeting and render M1GS targeting ineffective. These studies will lead to the generation of novel M1GS ribozymes with a better safety profile and provide insight into the development of M1GS ribozymes for anti-HBV therapy.

Fourth, future studies could explore the potential of M1GS in combination therapy for HBV. Many clinical trials are currently investigating the combination of nucleic acid-based therapeutics with approved antivirals, such as nucleos(t)ide analogs (NAs), with the aim of enhancing antiviral potency by combining strategies that act through different mechanisms. For example, the antisense oligonucleotide Bepirovirsen, which targets HBV mRNA through an RNase H-mediated mechanism, achieved a greater reduction in HBsAg when used alongside NAs (Table 1) [40]. M1GS shares a conceptual similarity with ASOs in that the GS-mediated sequence-specific base pairing targets the HBV mRNA before cleavage. This shared similarity suggests that M1GS could also benefit from combination therapy with NAs by synergistically reducing the RNA templates required for reverse transcription while NAs inhibit polymerase activity, limiting replenishment of the cccDNA reservoir. Although no direct data currently exist for M1GS combination therapy in HBV, the success of clinical trials of antisense-NA approaches provides a rationale for future investigation. Even if detailed clinical translation is premature for M1GS therapy at this stage, M1GS could serve as a powerful research tool for HBV biology through targeted knockdowns of specific HBV transcripts.

Finally, future studies could investigate pgRNA target sites beyond the overlapping regions conducted in the studies thus far. For example, overlapping regions involving other essential open reading frames, such as X ORF, could be investigated. The X gene encodes HBx, a multi-functional protein in HBV that promotes histone acetylation to decondense cccDNA and inhibits the tumor suppressor p53, often referred to as the “guardian of the genome” [88,89]. HBx is transcribed at low levels during chronic infection, and on its own, is not sufficient to trigger reactivation. However, HBx primes the virus, accelerating its transcriptional response when conditions such as cellular stress arise [88]. Experimental deletion of HBx has resulted in non-productive infection, highlighting its essential role in HBV replication [90]. Developing M1GS constructs beyond those currently tested could provide a broader antiviral strategy. The unique ability of M1GS to be efficiently engineered provides multiple lines of attack, allowing for a combination of M1GS-mediated knockdowns that collectively achieve broad and effective treatment.

## 10. Conclusions

In this review, we have discussed the gene-targeting capability of M1GS RNA, an engineered ribozyme derived from the catalytic RNA of bacterial RNase P. Using HBV infection as an example, we have examined the structure and function of M1GS ribozyme, its engineered form, and its potential antiviral applications. We explored how M1GS can cleave essential viral transcripts such as pgRNA and overlapping regions of the S and pre-S/L mRNAs, allowing for simultaneous interference with transcription, translation, and cccDNA replenishment through a single cleavage event. We have also explored future directions, including optimization of M1 RNA, compatibility with human RNase P proteins, improvements in delivery, and expansion of target sites. M1GS RNAs, characterized by their versatility and structure-based recognition, represent an exciting therapeutic prospect in gene-targeted antiviral therapy.

## Data Availability

Not applicable.
